# A novel approach to fit testing of the N95 respirator in real time in a clinical setting

**DOI:** 10.1186/1753-6561-5-S6-O34

**Published:** 2011-06-29

**Authors:** PP-L Or, JW-Y Chung, TK Wong

**Affiliations:** 1 School of Nursing, the Hong Kong Polytechnic University, Hong Kong, China; 2 Department of Health and Physical Education, The Hong Kong Institute of Education, Hong Kong, China; 3 The Hong Kong Polytechnic University, Hong Kong, Hong Kong, China

## Introduction / objectives

A fitted N95 respirator has been recommended, legislated in the USA and other countries. The conventional Portacount fit test method can only be used in a laboratory environment. It cannot deliver real-time measurements of face-seal leakage when the N95 respirator is in use in clinical settings. This study aimed to develop a method to evaluate N95 respiratory protection in real time, in a clinical setting.

## Methods

This research was divided into two stages. Stage 1 involved developing and validating a new fit test method to evaluate respirator protection. Stage 2 evaluated the performance of the new fit test method and the necessity to perform a “fit check”; Eighty-four subjects were selected for this study. They were divided randomly into four groups. The tests were conducted while the subjects were wearing N95 respirators in doing bedside nursing procedures.

## Results

Results from the work of Stage 1 showed that the new fit test method measured by the two spectrometers measured ambient particle concentration consistently. Results of Stage 2 showed significant differences among groups in perception of sensation after wearing N95 respirator in terms of the ease in talking (p=0.026). The mean of overall comfort perception of Group C was 4.24 (± 0.63), the highest among the four groups. Results of Stage 2 showed significant differences between Groups trained in performing the fit check and those who were untrained.

## Conclusion

A novel fit test method to evaluate N95 respirator protection was devised and tested in this study. If implemented, it could significantly reduce the risk of health care workers exposed to infectious diseases in clinical settings**.**

## Disclosure of interest

None declared.

